# Matrix metallopeptidase 9 contributes to the beginning of plaque and is a potential biomarker for the early identification of atherosclerosis in asymptomatic patients with diabetes

**DOI:** 10.3389/fendo.2024.1369369

**Published:** 2024-04-10

**Authors:** Bingli Liu, Liping Su, Sze Jie Loo, Yu Gao, Ester Khin, Xiaocen Kong, Rinkoo Dalan, Xiaofei Su, Kok-Onn Lee, Jianhua Ma, Lei Ye

**Affiliations:** ^1^ Department of Endocrinology, Nanjing First Hospital, Nanjing Medical University, Nanjing, China; ^2^ National Heart Research Institute Singapore, National Heart Centre Singapore, Singapore, Singapore; ^3^ Department of Cardiology, Ren Ji Hospital, School of Medicine, Shanghai Jiao Tong University, Shanghai, China; ^4^ Department of Endocrinology, Tan Tock Seng Hospital Lee Kong Chian School of Medicine Nanyang Technological University Singapore, Singapore, Singapore; ^5^ Division of Endocrinology, Department of Medicine, National University of Singapore, Singapore, Singapore

**Keywords:** atherosclerosis, type 2 diabetes, matrix metallopeptidase 9, inflammation, ischemic heart disease

## Abstract

**Aims:**

To determine the roles of matrix metallopeptidase-9 (MMP9) on human coronary artery smooth muscle cells (HCASMCs) *in vitro*, early beginning of atherosclerosis *in vivo* in diabetic mice, and drug naïve patients with diabetes.

**Methods:**

Active human MMP9 (act-hMMP9) was added to HCASMCs and the expressions of MCP-1, ICAM-1, and VCAM-1 were measured. Act-hMMP9 (n=16) or placebo (n=15) was administered to diabetic KK.Cg-*A^y^*/J (KK) mice. Carotid artery inflammation and atherosclerosis measurements were made at 2 and 10 weeks after treatment. An observational study of newly diagnosed drug naïve patients with type 2 diabetes mellitus (T2DM n=234) and healthy matched controls (n=41) was performed and patients had ultrasound of carotid arteries and some had coronary computed tomography angiogram for the assessment of atherosclerosis. Serum MMP9 was measured and its correlation with carotid artery or coronary artery plaques was determined.

**Results:**

*In vitro*, act-hMMP9 increased gene and protein expressions of MCP-1, ICAM-1, VCAM-1, and enhanced macrophage adhesion. Exogenous act-hMMP9 increased inflammation and initiated atherosclerosis in KK mice at 2 and 10 weeks: increased vessel wall thickness, lipid accumulation, and Galectin-3+ macrophage infiltration into the carotid arteries. In newly diagnosed T2DM patients, serum MMP9 correlated with carotid artery plaque size with a possible threshold cutoff point. In addition, serum MMP9 correlated with number of mixed plaques and grade of lumen stenosis in coronary arteries of patients with drug naïve T2DM.

**Conclusion:**

MMP9 may contribute to the initiation of atherosclerosis and may be a potential biomarker for the early identification of atherosclerosis in patients with diabetes.

**Clinical trial registration:**

https://clinicaltrials.gov, identifier NCT04424706.

## 1 Introduction

The consequences of atherosclerosis in the carotid and coronary arteries are obvious worldwide, especially in patients with diabetes. LDL-cholesterol is the only surrogate marker at present, and the identification of alternative or additional biomarkers may be useful for earlier diagnosis.

Multiple factors, including smoking, hypertension, hypercholesterolemia, and diabetes, contribute to atherosclerotic plaque formation in arteries (1). Although significant progress has been made in the understanding the development of atherosclerosis, such as endothelial activation, foam cell formation from lipids and macrophages, vascular smooth muscle cell (VSMC) proliferation, fatty streak formation, platelet activation, and thrombosis pathway activation, it is unclear what factors contribute to the initiation of plaques.

The matrix metalloproteinases (MMPs) are involved in plaque formation by degrading the extracellular matrix to weaken the arterial wall and cause cardiovascular remodeling (2–5). It has been shown that MMPs 2, 7, 8, 9, and 13 are involved in the plaque instability (2–5) and MMPs 2 and 9 expression are increased in advanced atherosclerotic lesions (5). MMP-9 is involved in atherosclerotic plaque rupture and tissue remodeling after a cardiac event (6). In addition, MMP-9 plasma levels correlate with myocardial infarction (MI) mortality, left ventricular (LV) remodeling and dysfunction in patients (7–11). Thus, these studies suggest that MMP9 may be directly involved in advanced atherosclerotic plaque and plaque instability and rupture and a potential diagnostic and prognostic biomarker of ischemic heart disease and heart failure.

However, the role of MMP9 in early atherosclerosis is not well studied. In the current study, we investigated the role of MMP9 in the early development of atherosclerosis in diabetes. We present a combination of *in vitro* experiments, *in vivo* animal, and human data in patients with type 2 diabetes mellitus (T2DM) and indicate that MMP9 may be potentially useful for the early detection of carotid artery plaque and coronary artery plaques in patients with diabetes.

## 2 Methods

### 2.1 *In vitro* studies

#### 2.1.1 MMP9 coculture with human coronary artery smooth muscle cells

Recombinant human matrix metallopeptidase 9 (hMMP9, RP75655) was purchased from Thermo Fisher. This was a 338-amino acid fragment (113-450) corresponding to the catalytic domain of the hMMP9 protein (act-hMMP9). Human coronary artery smooth muscle cells (HCASMCs, C0175C, Thermo Fisher) were maintained in smooth muscle cell growth medium (SMCGM) which was composed of M231 medium (M231500, Thermo Fisher) and smooth muscle growth supplement (S00725, Thermo Fisher). HCASMCs at passage 4 or 5 were used for experiments.

HCASMCs (5 x 10^4^/well) were seeded into 12-well plates and cultured in SMCGM. On day 1, medium was replaced with fresh SMCGM. On day 2, 200 ng/mL act-hMMP9 was added into designated wells for 24 h. On day 3, supernatants were collected, and cells were harvested either for quantitative RT-PCR (qRT-PCR) or Western Blot to quantify gene or protein expression levels of monocyte chemoattractant protein-1 (MCP-1), Vascular Cell Adhesion Molecule-1 (VCAM-1), and intercellular adhesion molecule-1 (ICAM-1).

Previous studies have shown that ERK1/2, P38MAPK, and NFkB signaling pathways are involved in the induction of MCP-1, ICAM-1 and VCAM-1 in different types of cells (12–22). Thus, in the current study, we employed respective inhibitors to determine whether act-MMP9 induced expressions of MCP-1, ICAM-1 and VCAM-1 were mediated by the three pathways. To determine signaling pathways through which act-hMMP9 induced expressions of MCP-1, VCAM1, and ICAM-1, 10 µM Losmapimod (Losma), an inhibitor for P38MAPK, 5 µM SCH772984 (SCH), an inhibitor for ERK1/2, and 100 µM pyrrolidine dithiocarbonate (PDTC), an inhibitor for NFĸB, were used. Inhibitors were added into designated wells 30 min before act-hMMP9 was added to inhibit respective signaling pathway. HCASMCs were harvested at 0, 30, 60, and 90 min to have protein isolated and assessed using Western Blot.

#### 2.1.2 Quantitative RT-PCR

Total RNA isolation and cDNA synthesis were performed as described previously (23–25). The PCR primers were listed in **Supplementary Table 1**. Endogenous GAPDH level was used as an internal control for normalization (23–25).

#### 2.1.3 Western blot

The protein expression levels of MCP-1 in the conditioned supernatant of HCSMCs, and VCAM-1, ICAM-1, phospho-ERK1/2 (pERK1/2), ERK1/2, phospho-P38MAPK (pP38MAPK), P38MAPK, phospho-NFĸB (pNFĸB), and NFĸB in HCASMCs were determined using Western Blot as described previously (26–28). The cell lysate was prepared using PhosphoSafe™ Extraction Reagent (Merck, Germany) and protein concentrations were determined using Bradford reagent (Bio-Rad Laboratories, USA). Proteins were separated, electrophoretically blotted onto nitrocellulose membranes, blocked, and incubated with respective primary antibodies (**Supplementary Table 2**) at 4°C overnight. Bound antibodies were detected with HRP-conjugated 2^nd^ antibodies (**Supplementary Table 2**) and visualized with a ChemiDoc™ MP Imaging System (Bio-Lab, USA) and Image Lab 5.1 software (Bio-Lab, USA).

#### 2.1.4 Human macrophages co-culture with HCASMCs

Human monocytes (MNs) were purchased from ATCC (TIB-202) and cultured in MN growth medium which was composed of RMPI-1640, 10% fetal bovine serum, and 0.05 mM 2-mercaptoethanol (21985023, Thermo Fisher) in a 5% CO_2_ incubator at 37°C. Cell cultured medium was changed every 2-3 days. To induce MN differentiation into macrophages, cells were cultured in RPMI160 medium supplemented with 10% FBS and 100 nM phorbol 12-myristate 13-acetate (PMA, 8139, Sigma-Aldrich) for 72 h (29). 15 ng/mL lipopolysaccharide (LPS) was added into cell cultured medium during the last 48 h to induce M1 macrophages. The cells were harvested and washed with DPBS three times and cultured in RPMI-1640 containing 10% FBS and 1 µg/mL 4,6-diamidino-2-phenylindole (DAPI) (Sigma, USA) for 24 h (26, 30). Finally, cells were washed with DPBS three times and used to co-culture with HCSMCs for adhesion assay.

For adhesion assay, HCASMCs (2 x 10^4^/well) were seeded on day 0 in SMCGM. On day 1, medium was replaced either with fresh SMCGM or fresh SMCGM supplemented with act-hMMP9 or in combination with Losma, SCH, or PDTC for 24 h. On day 2, 5 x 10^4^ DAPI labelled macrophages were added into designated wells for 24 h. On day 3, 100 µl of 4% PFA was added into each well for 5 min to fix cells. Then, non-adhesive cells were washed away using DPBS. Images under phase contrast and UV were taken using Olympus IX73 microscope and Cell Sens Standard software (Olympus, Japan). To quantitatively calculate adhesion, adhesion was graded in three categories: if a DAPI^+^ macrophage sits on a HCASMC, it would be graded as 2; if a DAPI^+^ macrophage sits next to a HCASMC, it would be graded as 1, otherwise it would be graded as 0. The mean adhesion grade was calculated = the sum of the numbers of macrophage x corresponding grade/total number of macrophages in each field.

### 2.2 *In vivo* studies of diabetic mice

#### 2.2.1 MMP9 and carotid artery atherosclerosis in diabetic mice

To investigate the role of MMP9 in early atherosclerosis development in diabetes, KK. Cg-*A^y^*/J mouse (KK mouse, Jackson Lab., USA), a T2DM mouse model, was used. The animal experimental protocol was approved by the Institutional Animal Care and Use Committee (IACUC) of the Singapore Health Services Pte Ltd, Singapore. All experimental and animal maintenance procedures were performed in accordance with the Animal Use Guidelines of the Singapore Health Services Pte Ltd and conformed to the guidelines from Directive 2010/63/EU of the European Parliament on the protection of animals used for scientific purposes.

KK mice at 3 months of age were screened for hyperglycemia by glucose tolerance test (GTT) as described (31–33). KK mice with hyperglycemia were randomly assigned into three animal groups: 1. KK mice with normal diet + saline injection (the ND + saline Group); 2. KK mice with high fat diet + saline injection (the HFD + saline Group); 3. KK mice with high fat diet + act-hMMP9 injection (the HFD + act-hMMP9 Group). The rodent high fat diet contains 60 Kcal% Fat (D12492, Research Diets, USA). Since no mouse act-MMP9 is available, act-hMMP9 was diluted at 1 µg/0.1 mL in saline for injection. 0.1 mL saline alone or containing 1 µg act-hMMP9 was retro-orbitally injected into mouse under anesthesia once every 2 days.

KK mice were euthanized 2 or 10 weeks after saline or act-hMMP9 treatment. Under anesthesia using inhaled 2% isoflurane, mice were intraperitoneally injected with 1000 U heparin and placed in supine position. After 5 min, the hearts were stopped by injection of 1 mL 10% KCl. Mice were perfused to remove blood. Briefly, 10 mL of 5 mM EDTA in PBS was slowly injected into left ventricle followed by 20 mL of ice-cold PBS. Next, 10 mL of ice-cold of 4% PFA in PBS was injected into the left ventricle and left for 10 min. Then, 10 mL of ice-cold 5% sucrose in PBS was injected into left ventricle to wash away the PFA. The left and right common carotid arteries (CCA) were isolated and embedded into OCT for cryo-sections.

#### 2.2.2 Histochemistry and immunohistochemistry assessments

##### 2.2.2.1 Vessel staining

To determine vessel wall thickness and inner circumference, cryo-sections of CCAs were stained with mouse anti-smooth muscle actin (SMA) conjugated with Cy3 (C6198, Sigma-Aldrich) and mouse anti-CD31 conjugated with FITC (sc-18916, Santa Cruz, USA). Vessels were imaged using Olympus IX73 microscope and Cell Sens Standard software (Olympus, Japan). Vessel wall thickness and inner circumference were calculated using photoshop (28, 34).

##### 2.2.2.2 Oil Red O staining

A stock Oil Red O (0.5%) isopropanol solution was prepared using Oil Red O (O-0625, Sigma-Aldrich) and stored in dark at 4°C. Oil red O working solution was freshly prepared by mixing 6 mL Oil Red O stock solution with 4 mL distilled H_2_O and filtered. Vessel samples were air-dried and fixed with 4% PFA at room temperature for 10 min and washed with PBS twice. Samples were soaked in 60% isopropanol for 1 min followed with Oil Red O working solution for 8 min. Samples were washed with 60% isopropanol, PBS, and air-dried. Then, samples were mounted with 90% glycerol for imaging.

##### 2.2.2.3 Sudan IV staining

A Sudan IV working solution was freshly prepared by mixing 0.1 g Sudan IV (198102, Scientific Laboratory Supplies Ltd, UK) in 50 mL acetone and 50 mL 70% ethanol and filtered. Vessel samples were air-dried and fixed with 4% PFA at room temperature for 10 min, washed with PBS, and rinsed in 70% ethanol for 30 sec. Samples were soaked in Sudan IV staining solution for 45 sec at 57°C, washed with 70% ethanol and distilled H_2_O, and air-dried. Then, samples were mounted with 90% glycerol for imaging.

##### 2.2.2.4 Galectin-3 (Mac-2) fluorescence staining

To determine the infiltration of macrophages into mice CCAs, fluorescence immunostaining for Galectin-3 (Mac-2) (SC-32790-FITC, Santa Cruz) to identify macrophages was performed as described previously (34–37).

### 2.3 MMP9 in patients with newly diagnosed diabetes

The study was performed in Nanjing First hospital, Nanjing, China. The study protocol was approved by the Ethics Committee of the hospital and written informed consent was obtained in all patients and subjects. All procedures conformed to the principles outlined in the Declaration of Helsinki. A total of 234 patients (M/F=181/53) with newly diagnosed drug naïve T2DM were recruited with the following inclusion criteria: 1). T2DM as defined by the World Health Organization in 1999; 2). aged between 18 and 80 years old; 3). completely anti-diabetic drug naive. Exclusion criteria were: 1). taking any anticoagulants, lipid-lowering medications, or plaque-stabilizing medications within the past three months; 2). any history of cardiovascular diseases, such as stroke, transient ischemic attack, myocardial infarction, unstable angina, coronary artery bypass grafting, percutaneous coronary intervention, or heart failure; 3). admitted acutely due to complications of diabetes, such as diabetic ketoacidosis, diabetic hyperosmolar nonketotic coma, or lactic acidosis; 4). presence of renal insufficiency (GFR < 60 mL/min/1.73 m^2^); 5). any other conditions that made them unsuitable to participate in the study, such as alcoholism and drug abuse.

All the newly diagnosed T2DM patients had carotid arteries ultrasound and were then assigned into 2 groups: patients with T2DM only without any detectable carotid artery plaques (the T2DM Group) and patients with T2DM and carotid artery plaque (the T2DM+CAP Group). Healthy subjects were recruited in a control group (N = 41, M/F = 22/19) defined by the following criteria: 1). normal oral glucose tolerance test (fasting glucose < 6.1 mmol/L and postprandial glucose < 7.8 mmol/L after 2 h); 2). aged between 18 and 80; 3) absence of carotid artery plaques; and 4) absence of any abnormal cardiac function on ECG and clinical examination. Coronary computed tomography angiogram (CTA) was performed on a subset of patients in the two T2DM groups: T2DM and T2DM+CAP.

#### 2.3.1 Clinical and blood biochemical measurements

Fasting venous blood samples were drawn and analyzed directly without freezing within 2 to 4 h of sampling at the Department of Endocrinology, Nanjing First Hospital, Nanjing, China. Serum total MMP9 (tMMP9) was measured using a Human MMP9 Quantikine ELISA kit (DMP900, R&D Systems, USA).

HbA1c was measured by high-performance liquid chromatography (HPLC) assay (Bio-Rad Laboratories, Inc. CA, USA). Plasma glucose was measured using the glucose oxidase method. Other blood biochemicals, including hemoglobin, alanine transaminase (ALT), aspartate aminotransferase (AST), alkaline phosphatase (ALP), blood urea nitrogen (BUN), creatinine (Cr), uric acid (UA), triglyceride (TG), total cholesterol (TC), high-density lipoprotein (HDL), and low-density lipoprotein (LDL), were measured as well.

#### 2.3.2 Total MMP9 and act-MMP9 measurement by Gelatin zymography

To measure serum act-MMP9, we combined tMMP9 results with gelatin zymography and calculated serum act-MMP9 concentration as: tMMP9 (ng/mL) x (act-MMP9 band intensity/tMMP9 band intensity), where the tMMP9 band intensity is the sum of pro-MMP9 band + act-MMP9 band intensities. The preparation of gelatin gel for zymography was performed as described (38). Briefly, fresh 4 mg/mL gelatin solution was prepared and mixed with 30% acrylamide, 1.5 M Tris (pH 8.8), distilled H_2_O, 10% SDS, 10% APS, and TEMED to make 9.5% acrylamide gel containing gelatin. Samples were mixed with 5 x non-reducing sample buffer and loaded into wells. The SDS gels were then washed with washing buffer, containing 2.5% Triton X-100, 50 mM Tris-HCl (pH 7.5), 5 mM CaCl_2_, and 1 µM ZnCl_2_ for 30 min twice and were incubated in incubation buffer containing 1% Triton x 100, Tris HCl 50 mM (pH 7.5), 5 mM CaCl_2_, 1 µM ZnCl_2_, and distilled H_2_O, for 10 min at 37°C with agitation. Finally, gels were incubated with fresh incubation buffer for 20 h at 37°C with agitation. On the 2^nd^ day, gels were stained with 0.5% Coomassie Blue staining solution for 30 min to 1 h. After rinsing with distilled H_2_O, gels were incubated with destaining solution containing 10% acetic acid with agitation until bands could be clearly seen.

#### 2.3.3 Carotid doppler ultrasound imaging acquisition and analysis

Carotid artery examinations were performed on a GE LOGIQ E9 color Doppler ultrasound diagnostic instrument equipped with an 8 ~ 10 MHz probe (GE). The left and right CCAs, external carotid arteries, and internal carotid arteries were scanned by the blinded clinicians who were experienced in carotid ultrasound. Two-dimensional images of the longitudinal and transverse sections were taken to detect and monitor the location, morphology (size and length), and number of carotid plaques of the patient (39, 40). The area sum of all carotid plaques was calculated for each patient (39, 40).

#### 2.3.4 Coronary computed tomography angiogram image acquisition and analysis

Coronary CTA was employed to detect, grade, and classify coronary atherosclerosis as described (41) using a Siemens Somatom Definition Flash Dual Source CT (Siemens, Germany) with pitch 0.20-0.48, collimation 32 * 2 mm * 0.6 mm, tube voltage 120 kV, and current 360 mA. Each data set was analyzed at least by 2 senior radiologists with more than 5 years of experience and level III training in coronary CTA. Quantitative plaque analysis was performed using semi-automated Circulation III software (Siemens, Germany) with manual correction of vessel contours if required (42, 43).

The main measures included plaque number, classification (calcified plaque, vulnerable plaque, and mixed plaque), coronary artery calcification scores (CACS), and degree of coronary luminal stenosis. Based on American College of Cardiology, coronary plaque is divided into three plaque types based on the CT values. fibro-lipid or fibro-fatty plaque: CT value < 60 HU; Calcified plaques: CT value ≥ 130 HU; Mixed plaques (mixed with the above two patches): 60 < CT value < 129 HU (44). CACS was calculated using the Shukun coronary artery calcification integral CT intelligent diagnostic system, which calculates coronary artery calcification integral using the Agatston integral method (45, 46). The luminal stenosis of coronary diseases was classically categorized into 4 degrees based on the percentage of cross-sectional area stenosis: 1% to 25%, 26% to 50%, 51% to 75%, and 76% to 100% (47, 48).

### 2.4 Statistical analysis

All statistical analyses were performed using SPSS (version 18.0). All data were presented as mean ± standard deviation (SD) or as median (IQR). P < 0.05 is considered to have a significant difference. The difference between 2 and 10 weeks within the same group was analyzed using paired T-Test. The difference between two groups was tested using independent T-Test. Overall differences between groups were tested for significance via one-way analysis of variance (ANOVA). When analysis of variance demonstrated a significant effect, *post hoc* analysis was performed using the Tukey test.

Binary Logistic regression and Pearson analysis (Spearman analysis in non-parametric variables) were performed to identify the correlation of parameters. A Chi-squared test was performed to compare the ratio differences between different groups. Receiver operating characteristic (ROC) analysis was performed to determine whether tMMP-9 was a good and reliable biomarker for diagnosis of arterial plaque.

## 3 Results

### 3.1 *In vitro* studies

#### 3.1.1 act-hMMP9 promoted MCP-1, ICAM-1, and VCAM-1 gene and protein expressions in HCASMCs

**Figure 1:**
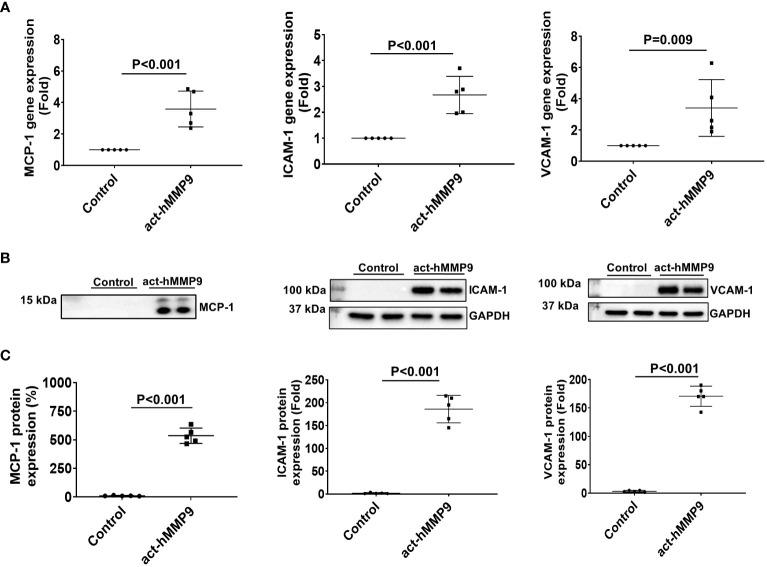
act-hMMP9 up-regulated expressions of MCP-1, ICAM-1, and VCAM-1 in human coronary artery smooth muscle cells (HCASMCs). **(A)** act-hMMP9 induced gene expressions of MCP-1, ICAM-1, and VCAM-1 in HCASMCs. **(B)** Representative images of Western Blot and **(C)** quantification of protein expressions of MCP-1 in the conditioned supernatant of HCASMCs, and ICAM-1, and VCAM-1 in HCASMCs induced by act-hMMP9. N = 5 biological replicates for each data. Values are presented as means ± SD. Independent T-Test.

act-hMMP9 at 200 ng/mL concentration significantly induced gene and protein expressions of MCP-1, ICAM-1, and VCAM-1 (**Figure 1**). Since MCP-1 attracts migration and infiltration of inflammatory cells like monocytes/macrophages to enhance inflammation (49), while ICAM-1 and VCAM-1 facilitate adhesion of inflammatory cells, including lymphocytes and monocytes (50, 51), these data indicate that act-hMMP9 induced up-regulation of MCP-1, ICAM-1, and VCAM_1 expression in HCASMCs, which may attract and promote adhesion of inflammatory cells, such as monocytes and macrophages.

#### 3.1.2 act-hMMP9 promoted MCP-1 expression in HCASMCs through ERK1/2/NFkB signaling pathway

**Figure 2:**
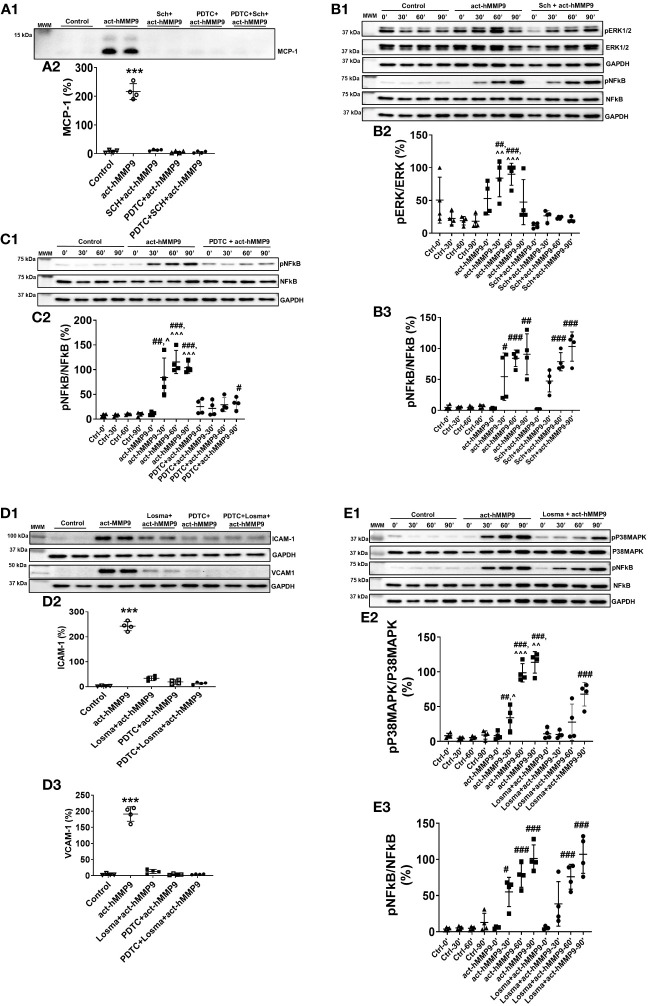
Western Blot for analyzing signaling pathways stimulated by act-hMMP9 in HCASMCs. Representative Western Blot image **(A1)** and quantification **(A2)** for MCP-1 protein expression in HCASMCs with or without act-MMP9 in the presence of Sch, an ERK1/2 inhibitor, PDTC, a NFkB inhibitor, or a combination of Sch and PDTC. **(B1)** Representative Western Blot image for protein expressions of pERK1/2, ERK1/2, pNFkB, and NFkB in HCASMCs as a function of time with or without act-hMMP9 in the presence of Sch. Quantification of pERK/ERK **(B2)** and pNFkB/NFkB **(B3)**. **(C1)** Representative Western Blot image for protein expressions of pNFkB and NFkB in HCASMCs as a function of time with or without act-hMMP9 in the presence of PDTC. **(C2)** Quantification of pNFkB/NFkB. **(D1)** Representative Western Blot images for ICAM-1 and VCAM-1 protein expressions in HCASMCs with or without act-MMP9 in the presence of Losma, a P38MAPK inhibitor, PDTC, or a combination of Losma and PDTC. Quantification of ICAM-1 **(D2)** and VCAM-1 **(D3)**. **(E1)** Representative Western Blot image for protein expressions of pP38MAPK, P38MAPK, pNFkB, and NFkB in HCASMCs as a function of time with or without act-hMMP9 in the presence of Losma. Quantification of pP38MAPK/P38MAPK **(E2)** and pNFkB/NFkB **(E3)**. N = 4 biological replicates for each data Values are presented as means ± SD. One-way ANOVA. **(A, D)** vs any other treatment, ***: p < 0.001. **(B, C, E)** vs Ctrl, #: p < 0.05, ##: p < 0.01, ###: p<0.001; vs Sch + act-hMMP9, PDTC + act-hMMP9, or Losma + act-hMMP9, ^: p < 0.05, ^^: p < 0.01, , ^^^: p < 0.001.

To test the potential key pathways through which act-hMMP9 induced up-regulation of MCP-1, ICAM-1, and VCAM-1, Western Blot analysis was performed. Western Blot showed that Sch (a potent ERK1/2 inhibitor), PDTC (a potent NFĸB inhibitor), or a combination of both inhibitors inhibited MCP-1 protein expression in the presence of act-hMMP9 (**Figure 2A**). Sch significantly inhibited ERK1/2 activity at 30 and 60 mins induced by act-hMMP9 (**Figures 2B1, B2**). However, act-hMMP9 induced NFkB activity was not inhibited by Sch (**Figures 2B1, B3**) and only PDTC inhibited pNFkB expression (**Figures 2C1, C2**). In addition, PDTC did not inhibit up-regulated activity of ERK1/2 in HCASMCs induced by act-hMMP9 (**Supplementary Figures 1A, B**), indicating that ERK1/2 is an upstream signaling molecule of NFkB. Collectively, these data indicates that act-hMMP9 activates MCP-1 expression through ERK1/2/NFkB signaling pathway and in addition to ERK1/2, act-hMMP9 can activate NFkB through other signaling pathways.

#### 3.1.3 act-hMMP9 promoted ICAM-1 and VCAM-1 expressions in HCASMCs through P38MAPK/NFkB signaling pathway

Western Blot analysis showed that Losma (a potent P38MAPK inhibitor), PDTC, or a combination of both inhibitors significantly reduced ICAM-1 and VCAM-1 protein expressions in the presence of act-hMMP9 (**Figure 2D**). Losma inhibited P38MAPK activity at 30, 60, and 90 min induced by act-hMMP9 (**Figures 2E1, E2**). However, Losma alone was unable to inhibit pNFkB expression (**Figures 2E1, 2E3**). In addition, PDTC did not inhibit up-regulated activity of P38MAPK in HCASMCs induced by act-hMMP9 (**Supplementary Figures 1C, D**), indicating that P38MAPK is also an upstream signaling molecule of NFkB. Collectively, these data suggest that act-hMMP9 activated ICAM-1 and VCAM-1 expressions through P38MAPK/NFkB signaling pathway.

Western Blot data suggest that act-hMMP9 can activate both ERK1/2 and P38MAPK through which lead to MCP-1, ICAM-1, and VCAM-1 expressions via activating NFkB. Thus, either a combination of ERK1/2 and P38MAPK inhibitors or a NFkB inhibitor is needed to inhibit act-hMMP9 biological function on HCASMCs.

#### 3.1.4 act-hMMP9 promoted macrophage adhesion to HCASMCs, which was inhibited by Losma and PDTC

**Figure 3:**
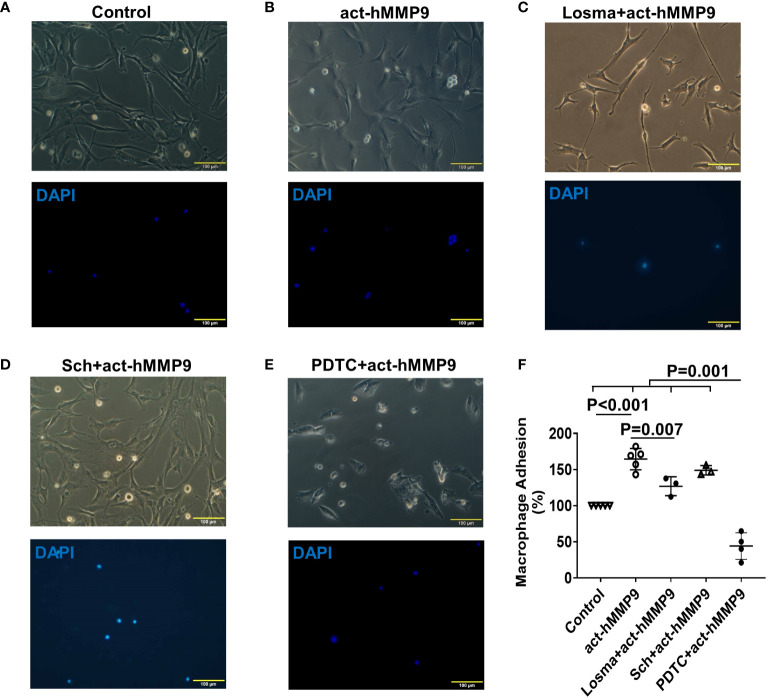
act-hMMP9 pretreated HCASMCs attracted adhesion of macrophages. HCASMCs were cultured in SMCGM only **(A)**, or SMCGM supplemented with 200 ng/mL act-hMMP9 **(B)**, or SMCGM supplemented with 10 µM Losma and 200 ng/mL act-hMMP9 **(C)**, or SMCGM supplemented with 5 µM Sch and 200 ng/mL act-hMMP9 **(D)**, or SMCGM supplemented with 100 µM PDTC and 200 ng/mL act-hMMP9 for 24 hours **(E)**. Then, activated macrophages were added into treated HCASMCs for 24 hours. **(F)** Quantification of macrophage adhesion to HCASMCs. N = 3 - 5 biological replicates for each data. Values are presented as means ± SD. One-way ANOVA.

Since act-hMMP9 promoted the protein expressions of adhesion molecules, i.e., ICAM-1 and VCAM-1, activated macrophages were co-cultured with HCASMCs. We found that the adhesion of macrophages to HCASMCs increased significantly by 64.4% as compared to the control (i.e., no act-hMMP9 treated cells were considered as 100%) (**Figures 3A, B, F**). Although Losma alone significantly reduced adhesion as compared with act-hMMP9 only treatment, it did not reach to the same level as the control cells (**Figures 3C, F**). Sch did not significantly reduce adhesion (**Figures 3D, F**) and only PDTC dramatically reduced adhesion to 44% of the control cells (**Figures 3E, F**). These results suggest that although both Losma and PDTC significantly reduced macrophage adhesion to HCASMCs and PDTC has the most inhibitory effect on macrophages adhesion to HCASMCs.

### 3.2 *In vivo* studies in KK diabetic mice

#### 3.2.1 act-hMMP9 injection increased wall thickness and reduced inner circumference of the carotid arteries

Human act-hMMP9 was injected into KK mice to determine the role of act-hMMP9 in the early development of atherosclerosis in diabetes. At week 2 after treatment, wall thicknesses of the CCAs increased significantly in KK mice of the HFD + act-hMMP9 Group as compared to the HFD + saline Group (**Supplementary Figures 2A, B**), while the inner circumferences of the CCAs were similar between the two groups (**Supplementary Figures 2A, C**).

At week 10 after treatment, although the wall thicknesses of the CCAs continued to further increase in both the HFD + saline and HFD + act-MMP9 Groups as compared to week 2 (p=0.012 and p=0.001, respectively), the wall thickness increase was significantly higher in the HFD + act-hMMP9 Group compared to the ND+ saline and HFD + saline Groups (**Supplementary Figures 2D, E**). The inner circumferences of the CCAs also showed significant changes – while the inner circumference in the HFD+ saline group increased significantly from week 2 to 10 (p=0.018), the HFD + act-hMMP9 Group did not have a statistically significant increase in the inner circumference (p=0.156) from weeks 2 to 10. Further confirming the severity of the atherosclerosis, the inner circumferences of the CCAs in the HFD + act-hMMP9 Group were significantly less than those of the ND + saline and HFD+ saline Groups at week 10 (**Supplementary Figure 2F**).

#### 3.2.2 act-hMMP9 injection increased lipid accumulation in the carotid arteries

**Figure 4:**
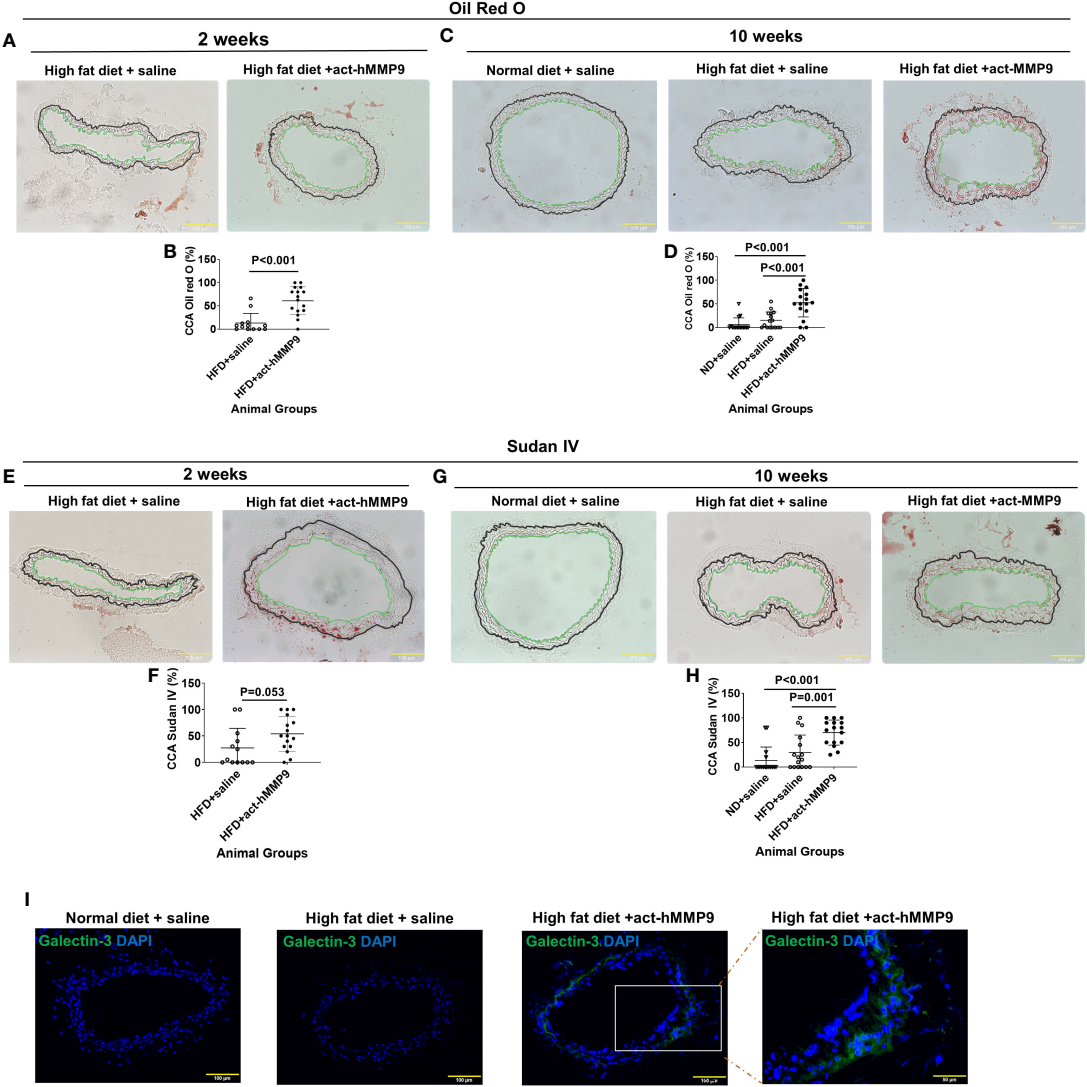
act-hMMP9 promoted pro-atherosclerosis in common carotid arteries (CCAs) of KK mice. **(A)** Oil Red O staining to visualize lipid accumulation in CCAs of KK mice at 2 weeks after treatment. **(B)** Quantification of Oil Red O in CCAs, which is presented as a percentage of Oil Red O area/total vessel wall area, in KK mice at 2 weeks after treatment. **(C)** Oil Red O staining to visualize lipid accumulation in CCAs of KK mice at 10 weeks after treatment. **(D)** Quantification of Oil Red O in CCAs of KK mice at 10 weeks after treatment. **(E)** Sudan IV staining to visualize lipid accumulation in CCAs of KK mice at 2 weeks after treatment. **(F)** Quantification of Sudan-IV in CCAs, which is presented as a percentage of Sudan-IV^+^ area/total vessel wall area, in KK mice at 2 weeks after treatment. **(G)** Sudan-IV staining to visualize lipid accumulation in CCAs of KK mice at 10 weeks after treatment. **(H)** Quantification of Sudan-IV in CCAs of KK mice at 10 weeks after treatment. The green line indicates the intima and the black line indicated external elastic lamina of the CCAs and the tissue located outside of the black line is adventitia. **(I)** Fluorescence staining for galectin-3 (Mac-2) protein expression to identify macrophages in CCAs of KK mice at 10 weeks after treatment. N = 13 or 16 CCAs which were isolated from 7 - 8 animals for each data. Values are presented as means ± SD. Panels **(B, F)** Independent T-Test; Panels **(D, H)** One-way ANOVA.

To assess the effect of act-hMMP9 on lipid accumulation in CCAs of KK mice, Oil Red O and Sudan IV stainings were performed (**Figure 4**). Oil Red O stained lipid was more commonly found in the CCAs of the HFD + act-hMMP9 Group as early as 2 weeks, compared to the HFD + saline Group (**Figures 4A, B**). This effect was also seen at week 10 after treatment (**Figures 4C, D**).

Sudan IV+ stained lipid had a similar trend of being more commonly found in the CCAs of the HFD + act-hMMP9 Group than in the HFD + saline Group at week 2 (**Figures 4E, F**), but reached clear statistical significance only at week 10 after treatment (**Figures 4G, H**). Collectively, these results suggest that act-hMMP9 injection stimulated lipid accumulation as early as 2 weeks in the CCAs of the KK mice.

#### 3.2.3 act-hMMP9 injection increased Galectin-3+ macrophage accumulation in the carotid arteries

Fluorescence immunostaining of Galectin-3, a marker of activated macrophages involved in atherosclerotic plaque progression (52), showed that Galectin-3+ cells were not found in 16 CCAs of the mice in the ND + saline or HFD + saline Groups, but were more commonly found in CCAs (10 out of 16 CCAs) of the HFD + act-hMMP9 Group, which were significantly higher than those of the ND + saline and HFD + saline Groups (p<0.001, among 3 groups by Chi-squared tests) (**Figure 4I**). These results suggest that act-hMMP9 injection resulted in Galectin-3+ macrophage accumulation in the CCAs of KK mice in the HFD + act-hMMP9 Group.

C) Studies in newly Diagnosed drug naïve T2DM patients

Serum total MMP9 (tMMP9) and act-MMP9 were higher in patients with T2DM and associated positively with severity of carotid artery plaque

A total of 275 subjects (41 healthy controls, 107 newly diagnosed T2DM with normal carotid arteries, and 127 newly diagnosed T2DM with carotid artery plaques seen on ultrasound) were recruited in this clinical study (**Supplementary Table 1**). Patients with T2DM had significantly higher BMI, FBG, HbA1c, TG, ALT, ALP, and lower HDL than those in the healthy control Group. In addition, LDL was significantly higher in the T2DM + CAP Group than the T2DM Group (**Table 1**).

**Table 1:** Patient characteristics.

	Healthy control	T2DM	T2DM+CAP	P value
**Case (number)**	n=41	n=107	n=127	
**Gender (Male,n,%)**	22 (53.7%)	70 (65.4%)	89 (70.1%)	0.155
**Smoking (n,%)**	3 (7.3%)	12 (11.2%)	20 (15.7%)	0.310
**Age (years)**	47.02±9.66	48.00±11.01	50.53±10.57	0.084
**BMI (Kg/m** ^2^)	23.38±2.19	25.74±3.84**	25.77±3.55**	<0.01
**FBG (mmol/L)**	5.69±0.42	11.99±3.88**	11.58±3.61**	<0.01
**History of diabetes (years)**	0	0.46±1.12**	0.54±1.27**	<0.01
**HbA1c (%)**	5.19±0.32	10.07±1.89**	10.19±1.99**	<0.01
**Hypertesnion (n,%)**	1 (2.4%)	38 (35.8%)**	48 (38.1%)**	<0.01
**Haemoglobin (g/L)**	141.95±12.5	143.31±13.88	144.52±12.27	0.512
**ALT (U/L)**	21.98±14.42	34.29±26.35**	32.51±25.56*	0.017
**AST (U/L)**	19.80±7.73	21.44±14.66	21.15±15.00	0.817
**ALP (U/L)**	76.12±22.94	95.87±26.54**	95.48±27.77**	<0.01
**BUN (mmol/L)**	5.05±1.26	5.51±1.33	5.65±1.55	0.069
**Cr (µmol/L)**	64.84±13.7	60.35±12.85	63.93±15.79	0.090
**UA (µmol/L)**	319.8±83.86	294.32±76.6	302.35±88.14	0.257
**TG (mmol/L)**	1.40±1.19	2.50±3.17*	2.41±1.86*	0.035
**TC (mmol/L)**	5.04±1.28	4.94±1.03	5.65±4.92	0.264
**HDL (mmol/L)**	1.29±0.30	1.12±0.31**	1.11±0.28**	<0.01
**LDL (mmol/L)**	2.81±0.82	2.57±0.74	2.84±0.97^&^	0.059

* : vs Healthy control, p<0.05; ** :vs Healthy control, p<0.01. & : vs T2DM, p<0.05.

T2DM, type 2 diabetes mellitus; CAP, carotid artery plaque.

BMI, Body mass index; FBG, Fasting blood glucose; HbA1c, Hemoglobin A1c; ALT, Alanine transaminase; AST, Aspartate transaminase; ALP, Alkaline phosphatase; BUN, Blood urea nitrogen; Cr, Creatinine; UA, Uric acid; TG, Triglyceride; TC, Total cholesterol; HDL, High density lipoprotein; LDL, Low density lipoprotein.

**Figure 5:**
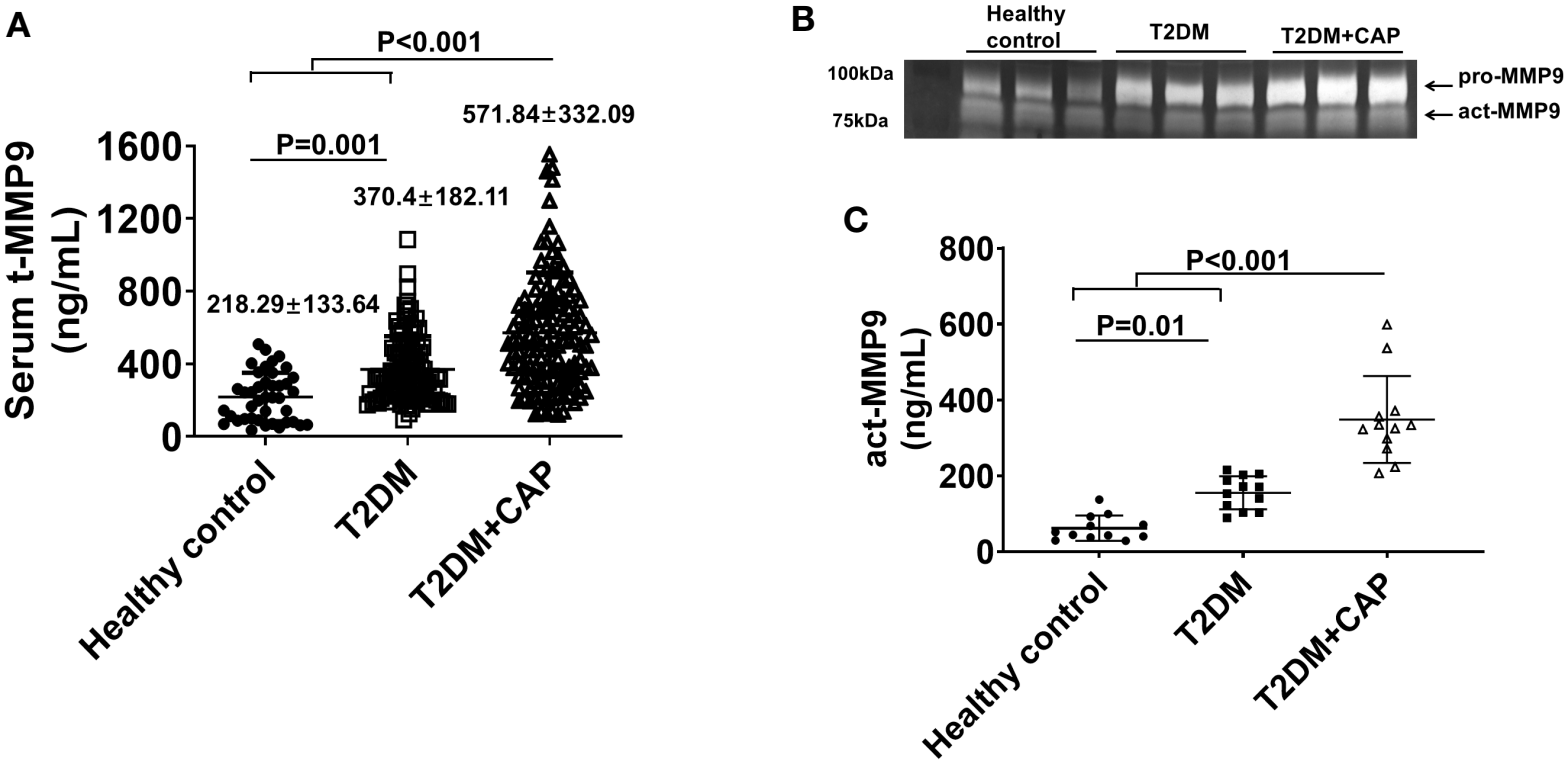
Characterization of serum MMP9 in 275 subjects recruited in this study. **(A)** A scatter dot plot of serum tMMP9, measured via human MMP9 ELISA kit, in the healthy control subjects and T2DM and T2DM+CAP patients. (Healthy control = 41, T2DM = 107, and T2DM + CAP = 127). **(B)** Representative zymography imaging for analyzing serum MMP9 in the healthy control subjects and T2DM and T2DM+CAP patients. **(C)** Quantification of serum act-MMP9 in the healthy control subjects and T2DM and T2DM+CAP patients. The act-MMP9 was calculated as a ratio of act-MMP9/tMMP9 x tMMP9, which was measured via human MMP9 ELISA kit. N = 12 each for the healthy control, T2DM, and T2DM + CAP Groups. Values are presented as means ± SD. One-way ANOVA.

The mean serum tMMP9 (218.29 ± 133.64 ng/mL) in the healthy control Group was significantly lower than that in the T2DM group (370.4 ± 182.11 ng/mL, p = 0.001) and the T2DM + CAP Group (571.84 ± 332.09 ng/mL, p < 0.001) (**Figure 5A**). The mean serum tMMP9 in the T2DM + CAP Group was significantly higher than that in the T2DM Group (p < 0.001). Linear correlation analysis suggested that there was a linear correlation between the serum tMMP-9 and area of atherosclerotic plaque in the carotid arteries (Pearson correlation coefficient was 0.414, p < 0.01) of the T2DM + CAP Group, suggesting that the serum tMMP9 may be associated with the severity of carotid artery atherosclerosis in newly diagnosed T2DM patients.

As the human MMP9 ELISA only measures tMMP9 which includes proactive MMP9 (pro-MMP9) and act-MMP9, zymography was performed to visualize and quantify act-MMP9. Zymography of patient serum showed that the intensities of pro-MMP9 and act-MMP9 bands were associated with severity of carotid artery plaque (**Figure 5B**). The act-MMP9 level in the T2DM + CAP Group was the highest as compared with those in the healthy control and T2DM Groups (**Figure 5C**). These results suggest that both serum tMMP9 and act-MMP9 levels increased in diabetes and are associated with severity of carotid artery plaque in diabetes.

#### 3.2.4 Both cholesterol and tMMP9 were independent contributory factors of carotid artery plaque formation

To determine which factor contributes to carotid artery plaque formation, multiple linear regression analyses were performed in patients with newly diagnosed T2DM + CAP. Analysis showed that only Cholesterol and tMMP9 levels remained significant in the stepwise regression analysis. The standardized regression coefficients were 14.797 (t = 13.075, P < 0.01) and 0.109 (t = 5.290, P < 0.01), respectively, suggesting that cholesterol and tMMP9 are two independent factors contributing to the carotid artery plaque formation.

#### 3.2.5 tMMP9 correlated with the number of mixed plaques and grade of lumen stenosis in coronary arteries

Coronary CTA was performed in patients who gave informed consent to have coronary CTA in the T2DM (n=15) and T2DM + CAP (n=47) Groups (**Table 2**). 13 out of 15 patients in the T2DM Group and 43 out of 47 patients in the T2DM + CAP Group had coronary artery plaques detected by coronary CTA. T2DM+CAP Group had trends of increased coronary CTA calcification score, numbers of plaques, calcified plaques, mixed plaques and non-calcified plaques, and grade of coronary luminal stenosis, but they were not significantly different from the T2DM Group.

**Table 2:** Patient characteristics who had coronary CTA.

	T2DM	T2DM+CAP	P value
**Case (number)**	n = 15	n = 47	
**Gender (M/F)**	10/5	35/12	0.555
**Age (years)**	55.6±9.3	52.32±10.29	0.276
**BMI (Kg/m2)**	24.28±3.5	26.08±4.34	0.151
**FBG (mmol/l)**	10.34±2.89	12.19±3.53	0.071
**History of diabetes** **(years)**	0.13±0.52	0.09±0.35	0.683
**HbA1c (%)**	9.91±1.94	10.17±1.9	0.643
**Hypertesnion** **(case)**	n = 7	n = 23	0.878
**Haemoglobin (g/L)**	143.33±14.67	143.57±13.46	0.953
**ALT (U/L)**	32.03±27.23	36.12±43.24	0.732
**AST (U/L)**	19.03±13.75	22.54±21.94	0.569
**ALP (U/L)**	107.81±17.35	101.41±27.75	0.404
**BUN (mmol/L)**	5.67±1.14	5.75±1.72	0.875
**Cr (µmol/L)**	55.86±9.42	64.35±17.92	0.085
**UA (µmol/L)**	279.63±83.1	317.03±93.16	0.170
**TG (mmol/L)**	1.76±0.69	2.71±2.48	0.149
**TC (mmol/L)**	4.82±1.03	5.41±1.03	0.057
**HDL (mmol/L)**	1.18±0.27	1.07±0.23	0.129
**LDL (mmol/L)**	2.29±0.77	3.06±1.05^&^	0.013
**Serum t-MMP9**	293.87±126.37	529.38±355.71^&^	0.017
**Coronary CTA** **calcification score**	18.05±26.43	50.59±91.31	0.180
**Number of calcified** **plaques**	0.66±1.23	1.55±2.43	0.181
**Number of mixed** **plaques**	0.47±1.06	1.06±1.51	0.161
**Number of non-** **calcified plaques**	1.53±1.64	2.19±1.74	0.201
**Total number of** **plaques**	2.67±2.79	4.81±4.58	0.093
**Grade of coronary** **lumen stenosis**	X^2^=8.070		0.089

&: vs T2DM, p<0.05.

T2DM, type 2 diabetes mellitus; CAP, carotid artery plaque.

BMI, Body mass index; FBG, Fasting blood glucose; HbA1c, Hemoglobin A1c; ALT, Alanine transaminase; AST, Aspartate transaminase; ALP, Alkaline phosphatase; BUN, Blood urea nitrogen; Cr, Creatinine; UA, Uric acid; TG, Triglyceride; TC, Total cholesterol; HDL, High density lipoprotein; LDL, Low density lipoprotein; MMP9, Matrix metallopeptidase 9; TIMP1, tissue inhibitor of metalloproteinase-1; TIMP2, tissue inhibitor of metalloproteinase-2.

**Figure 6:**
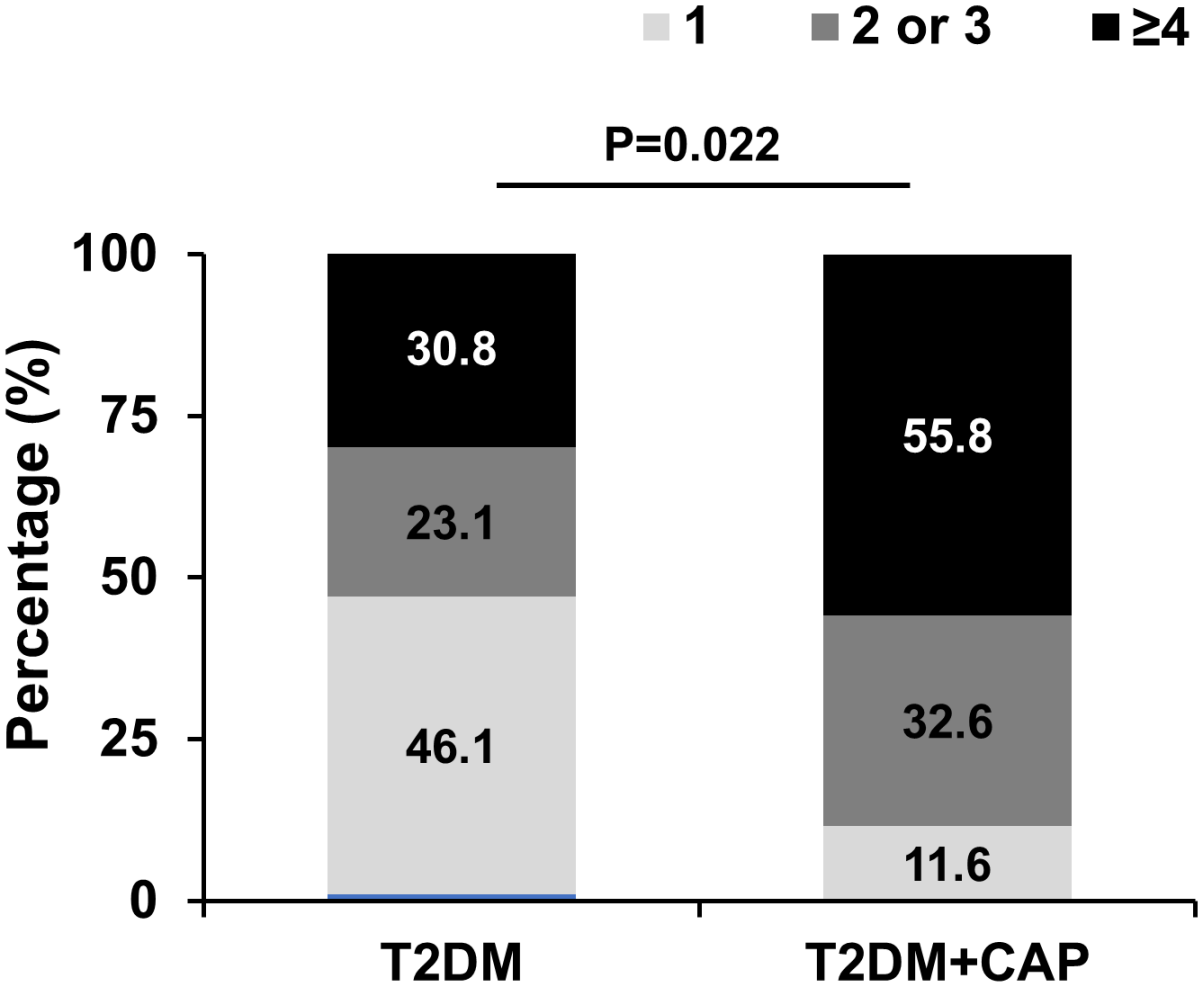
T2DM + CAP Group had a greater number of plaques in the coronary arteries as detected via coronary CTA. The number of plaques ≥ 4 in coronary arteries was significantly higher in the T2DM+CAP Group than that in the T2DM Group as analyzed using Chi-squared test: p=0.022. (N = 13 for the T2DM Group and N = 43 for the T2DM + CAP Group).

Spearman correlation analysis showed that tMMP9 correlated with number of mixed plaques and grade of lumen stenosis in coronary arteries (**Table 3**). There was no correlation between tMMP-9 and the number of calcified plaques or the calcification score. Furthermore, the total numbers of plaques (n ≤ 3 = 69.3% and n ≥ 4 = 30.8%) in the T2DM Group were significantly less than those (n ≤ 3 = 44.2% and ≥ 4 = 55.8%) in the T2DM + CAP Group (**Figure 6**).

**Table 3:** Spearman correlation analysis of tMMP9 and CTA parameters.

	Serum t-MMP9 (ng/ml)	Coronary Artery Calcium Scoring	Number of calcified plaques	Number of mixed plaques	Number of non-calcified plaques	Total number of plaque	Grade of coronary artery lumen stenosis^1^
Pearson correlation	1.000	0.211	-0.031	0.316	0.226	0.230	0.321
P value	/	0.103	0.815	0.013	0.079	0.075	0.012
Case (number)	62	62	62	62	62	62	62

^1^Grade of coronary lumen stenosis: 1: no stenosis; 2: stenosis < 25%; 3: 25% < stenosis < 500%; 4: 50% < stenosis < 75%; 5: stenosis: > 75.0%.

#### 3.2.6 tMMP9 is a potential biomarker for assessing arterial plaque.

**Figure 7:**
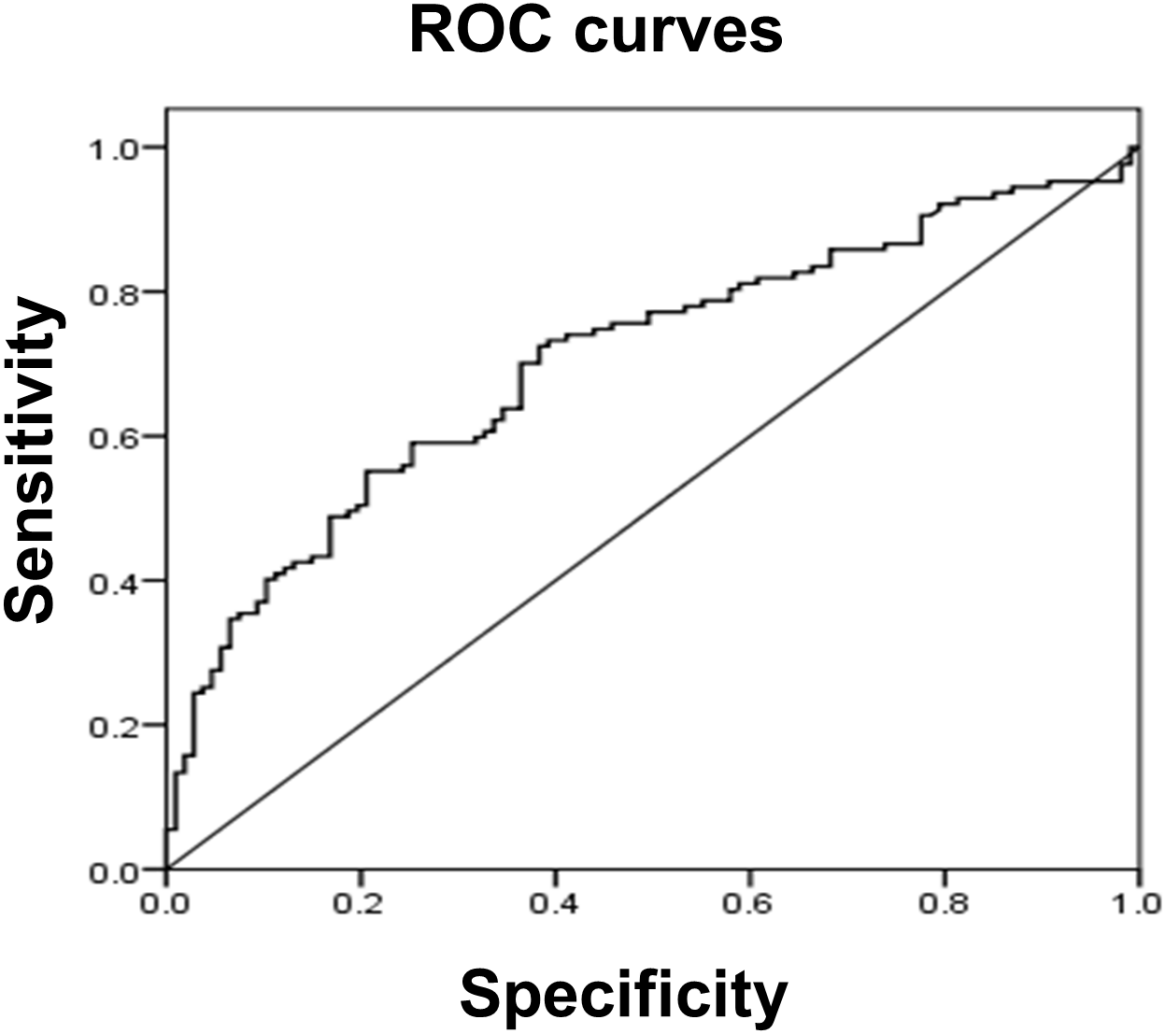
Receiver operating characteristic (ROC) curve analysis suggested that serum tMMP9 is a good and reliable biomarker for predicting arterial plaque in drug naïve T2DM with asymptotic atherosclerosis. The area under the ROC curve was 0.704 (p<0.01). The best cutoff point of plasma MMP-9 level was 498.60 ng/mL and its sensitivity and specificity were 0.551 and 0.794, respectively.

Since serum tMMP9 is an independent factor that contributes to carotid artery plaque in patients with naïve T2DM, receiver operating characteristic (ROC) analysis was performed to assess tMMP9 as a biomarker for early diagnosis of arterial plaque (**Figure 7**). ROC analysis showed that area under curve (AUC) area was 0.704 (p<0.01) and the best cutoff point of serum tMMP9 level was 498.60 ng/mL. Its sensitivity and specificity were 0.551 and 0.794, respectively, suggesting that tMMP9 may be a useful marker for predicting arterial plaque.

## 4 Discussion

There is an unmet need for biomarkers for atherosclerosis in patients with Diabetes for early diagnosis and intensive treatment. In the current study, we have presented evidence for the direct involvement of MMP9 in the atherosclerosis process, and also correlated this with *in vivo* evidence from a mouse model of T2DM. We found that *in vitro* act-hMMP9 up-regulated expressions of MCP-1, ICAM-1, and VCAM-1 in HCASMCs and induced adhesion of macrophages to HCASMCs through ERK1/2/NFkB and P38MAPK/NFkB signaling pathways, *in vivo* act-hMMP9 increased wall thickness and lipid and macrophage accumulation in CCAs of KK mice. Finally, we have measured serum MMP9 in healthy controls and in T2DM patients and found that serum tMMP9 level correlated with carotid artery plaque size and number of mixed plaques, plaque stability, and grade of lumen stenosis in coronary arteries. Our study suggests that MMP9 may be a potential biomarker for the earlier detection of carotid artery and coronary artery plaques in patients with diabetes.

MMP9, also called gelatinase B, is an enzyme that belongs to the zinc-metalloproteinases family involved in the degradation of the extracellular matrix. It is associated with numerous pathological diseases, such as rheumatoid arthritis, cancer, atrial fibrillation, aortic aneurysm, and atherosclerosis etc. (53–57). Studies have shown that MMP9 alters post-injury vascular remodeling by inducing endothelial cell apoptosis and activation (58) and is required for SMC migration (59, 60) and proliferation (59). In diabetes, hyperglycemia induces the production of reactive oxygen species, which results in the increased activity of MMP9 (61). MMP9 attracts monocyte/macrophage infiltration into plaque (62). Elevated MMP9 level is found in rupture-prone shoulder regions of human vulnerable atherosclerotic plaques (63). Thus, it has been suggested that MMP9 is a marker of carotid plaque instability (64) or plaque rupture (65, 66) and a predictor of adverse clinical outcome in patients with acute coronary syndrome (66). Although it is known that MMP9 is involved in the progression of plaque and helps predict plaque instability, it is unclear about its role in the early development of atherosclerosis for early risk stratification, especially in patients with diabetes.

In the current study, we found that *in vitro* act-hMMP9 up-regulated expressions of MCP-1, ICAM-1,a nd VCAM-1 in HCASMCs and enhanced adhesion of macrophages to HCASMCs. actMMP9 activates both ERK1/2 and P38MAPK signaling pathways which converge at NFkB and lead to downstream (ICAM1, VCAM1, and MCP1) upregulation. A combination of these inhibitors may needed to achieve the most robust inhibitory effect of act-MMP9 on HCASMCs in the long term. *In vivo* act-hMMP9 increased vessel wall thickness and lipid accumulation, enhanced macrophage infiltration, reduced vessel inner circumference of CCAs in KK mice fed with HFD. However, plaque was not found in CCAs up to 10 weeks after act-hMMP9 injection into KK mice, indicating that act-hMMP9 is involved in the early development of plaques in diabetes, as all of these observations are key early steps in plaque development (67).

Furthermore, we explored the associations of MMP9 with carotid artery and coronary artery plaques. Linear correlation analysis showed a linear correlation between serum tMMP9 level and area of atherosclerotic plaque in the carotid arteries of patients with newly diagnosed T2DM. The ROC analysis suggested that tMMP9 is a good and reliable biomarker for early diagnosis of arterial plaque and the best cutoff point of serum t-MMP9 level was 498.60 ng/mL. This level is about 38.5 folds of that reported by Somuncu et al., who showed that patients with MI, who had MMP-9 plasma levels above 12.92 ng/mL at the time of hospital admission, had 3.5-fold higher odds for cardiovascular mortality and increased risk for advanced heart failure compared to patients with lower MMP-9 level (8). This different MMP9 level in plasma could be due to the different ELISA kits used, as the MMP9 level in our study falls in the same range as Zhong’s study (68), in which used the same ELISA kit as ours.

Due to the anatomic position, coronary artery plaques are often omitted in asymptomatic patients and cause heart attacks which are associated with high mortality. A cost-effective biomarker is needed for early screening of coronary artery plaque in asymptomatic patients. Thus, we assessed the tMMP9 as a potential biomarker for detecting coronary artery plaque. Coronary CTA examination found 13 out of 15 patients in the T2DM Group and 43 out of 47 patients in the T2DM+CAP Group had plaques in coronary arteries. The atherosclerosis is more severe in the T2DM + CAP Group as compared with the T2DM Group. About 46% of the patients in the T2DM Group had only 1 plaque, while > 55% of patients in the T2DM+ CAP Group had at least 4 plaques detected by coronary CTA. Spearman correlation analysis showed that t-MMP9 correlated with the number of mixed plaques and grade of lumen stenosis in coronary arteries. It is not surprising tMMP9 correlated with coronary plaque stability as MMP9 has been shown as a marker of plaque instability (64) or plaque rupture (65, 66). However, tMMP9 also correlated the number of mixed plaques and grade of lumen stenosis in coronary arteries, indicating that tMMP9 is associated with severity of coronary artery atherosclerosis.

Our study aligns with early studies showing that MMP-9 plasma levels correlate with MI mortality, LV remodeling and dysfunction in patients (7–11). Even the transcriptional activity of MMP9 is associated with the severity of heart failure (69). However, all these studies targeted MMP9 at later stage of ischemic heart disease or heart failure, while we explored the potential of MMP9 as a useful biomarker for early detection of carotid artery plaques and coronary artery plaques in drug naïve patients with diabetes with subclinical atherosclerotic vascular disease.

A limitation of this study is that we did not perform *in vivo* experiments to investigate the potential mechanism of act-hMMP9 on lipid and macrophage accumulations in carotid arteries of KK mice. A transgenic mouse model to over-express actMMP9 peptide in SMCs shall be created to further address this.

**Figure 8:**
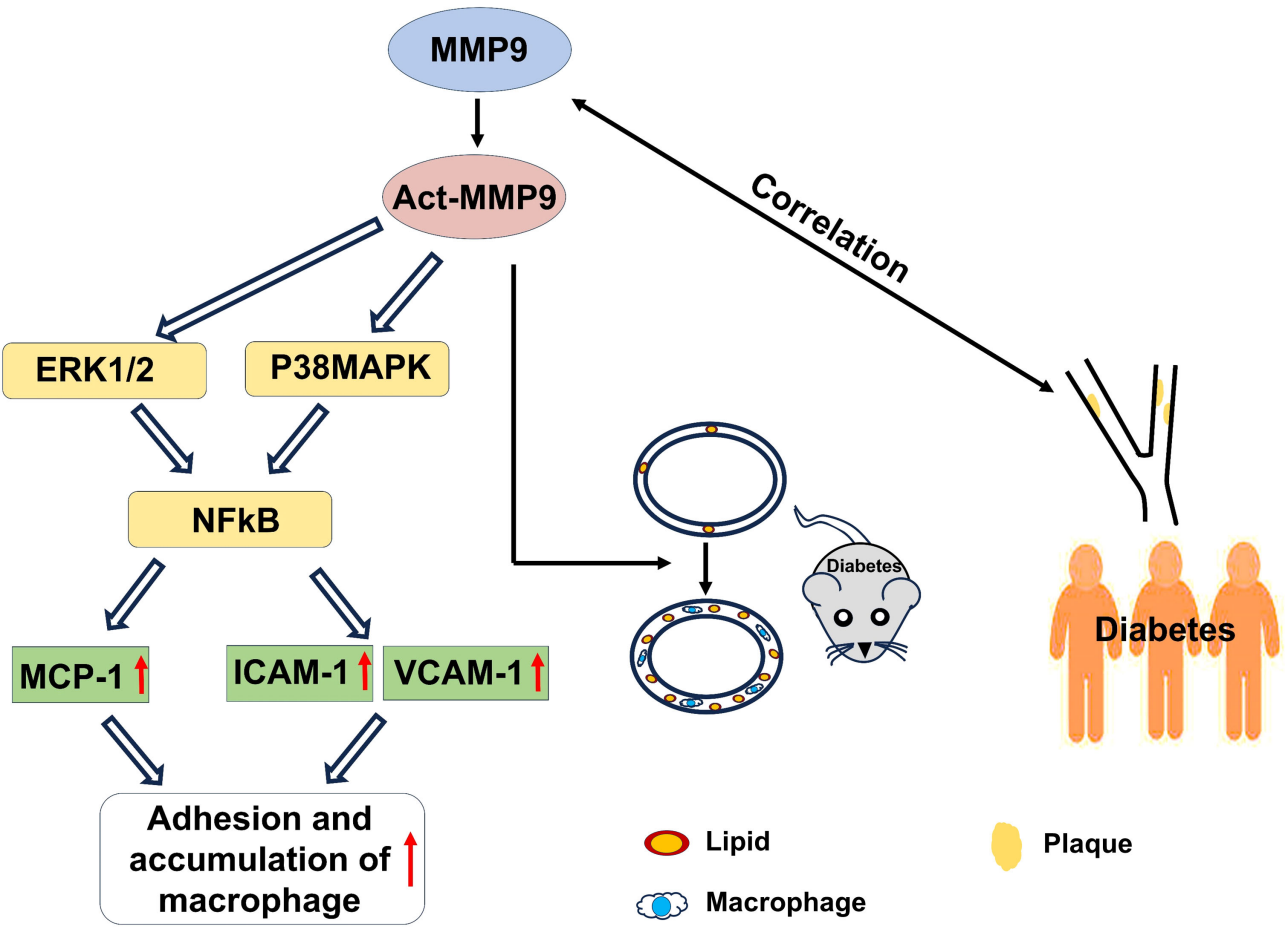
A graphic illustration to summary the findings of the study. *In vitro*, act-MMP9 increased the expressions of MCP-1, ICAM-1, VCAM-1 in HCASMCs, and enhanced macrophage adhesion. *In vivo*, act-MMP9 increased vessel wall thickness, lipid and macrophage accumulation in carotid arteries of KK mice. In newly diagnosed T2DM patients, serum MMP9 correlated with carotid artery plaque size, number of mixed plaques and grade of lumen stenosis in coronary arteries of patients with drug naïve T2DM.

In conclusion, act-hMMP9 stimulated up-regulated expressions of MCP-1, ICAM1,a nd VCAM1 in HCASMCs and enhanced adhesion of macrophages to HCASMCs *in vitro*, induced pro-atherosclerotic condition in carotid arteries of KK mice *in vivo*, and clinically serum tMMP9 level is correlated with area of carotid artery plaque and the number of mixed plaques, plaque stability, and grade of lumen stenosis in coronary arteries (**Figure 8**). Thus, tMMP9 may be a potentially useful biomarker for early detection of carotid artery plaques and coronary artery plaques in patients with subclinical atherosclerotic vascular disease.

## Data availability statement

The original contributions presented in the study are included in the article/**Supplementary Material**. Further inquiries can be directed to the corresponding authors.

## Ethics statement

The clinical study was performed in Nanjing First hospital, Nanjing, China. The study protocol was approved by Ethics Committee of the hospital and written informed consent was obtained in all patients and subjects. All procedures conformed to the principles outlined in the Declaration of Helsinki. The studies were conducted in accordance with the local legislation and institutional requirements. The participants provided their written informed consent to participate in this study. The animal experimental protocol was approved by the Institutional Animal Care and Use Committee (IACUC) of the Singapore Health Services Pte Ltd, Singapore. The study was conducted in accordance with the local legislation and institutional requirements. No potentially identifiable images or data are presented in this study.

## Author contributions

BL: Data curation, Formal analysis, Investigation, Methodology, Software, Writing – review & editing. LS: Data curation, Formal analysis, Investigation, Methodology, Software, Writing – review & editing. SL: Data curation, Formal analysis, Investigation, Methodology, Software, Writing – review & editing. YG: Data curation, Formal analysis, Investigation, Methodology, Software, Writing – review & editing. EK: Data curation, Formal analysis, Investigation, Methodology, Software, Writing – review & editing. XK: Data curation, Formal analysis, Investigation, Methodology, Software, Writing – review & editing. RD: Resources, Writing – review & editing. XS: Investigation, Methodology, Project administration, Resources, Supervision, Visualization, Writing – review & editing. KL: Writing – review & editing. JM: Conceptualization, Funding acquisition, Investigation, Project administration, Resources, Supervision, Validation, Visualization, Writing – review & editing. LY: Conceptualization, Data curation, Formal analysis, Funding acquisition, Investigation, Methodology, Project administration, Resources, Software, Supervision, Validation, Visualization, Writing – original draft, Writing – review & editing.
