# Supplementary material for: Matrix metallopeptidase 9 contributes to the beginning of plaque and is a potential biomarker for the early identification of atherosclerosis in asymptomatic patients with diabetes
Source: Front Endocrinol (Lausanne). 2024 Apr 10;15:1369369. doi: 10.3389/fendo.2024.1369369 (PMC11039961; doi:10.3389/fendo.2024.1369369)
Supplement: Supplementary file 1 [file DataSheet_1.pdf]

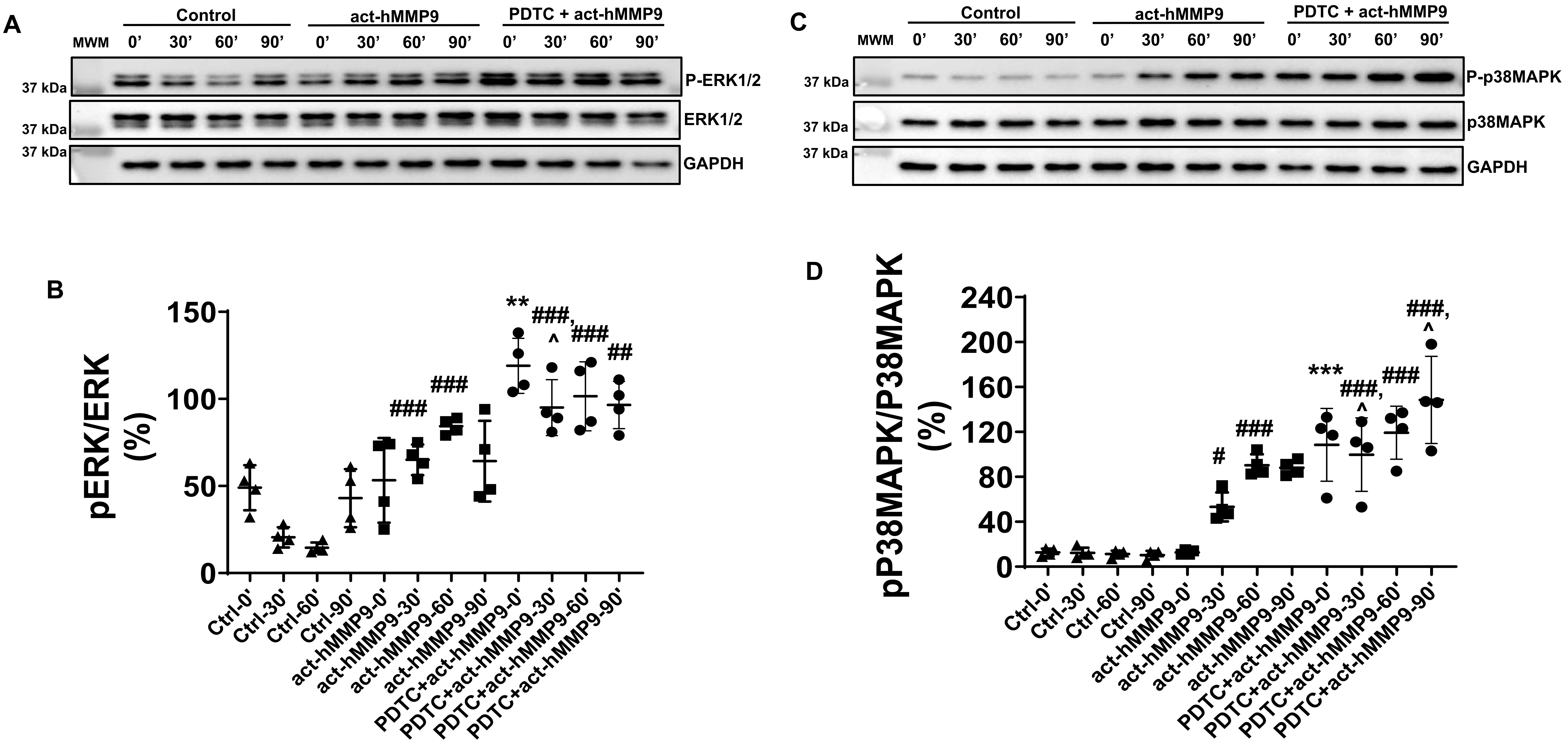

Supplemental Figure 1. Western Blot for analyzing ERK1/2 and P38MAPK signaling pathways inhibited by PDTC in the presence of act-hMMP9. Representative Western Blot image **(A)** and quantification **(B)** for P-ERK1/2 and ERK1/2 protein expressions in HCASMCs with or without PDTC and/or act-hMMP9. Representative Western Blot image **(C)** and quantification **(D)** for P-p38MAPK and p38MAPK protein expressions in HCASMCs with or without PDTC and/or act-hMMP9. N = 4 biological replicates for each data Values are presented as means ± SD. One-way ANOVA. Vs Ctrl and act-hMMP9 treatment: **: p<0.01 and ***: p < 0.001; Vs Ctrl, #: p < 0.05, ##: p < 0.01, ###: p<0.001; vs act-hMMP9 treatment: ^: p < 0.05.

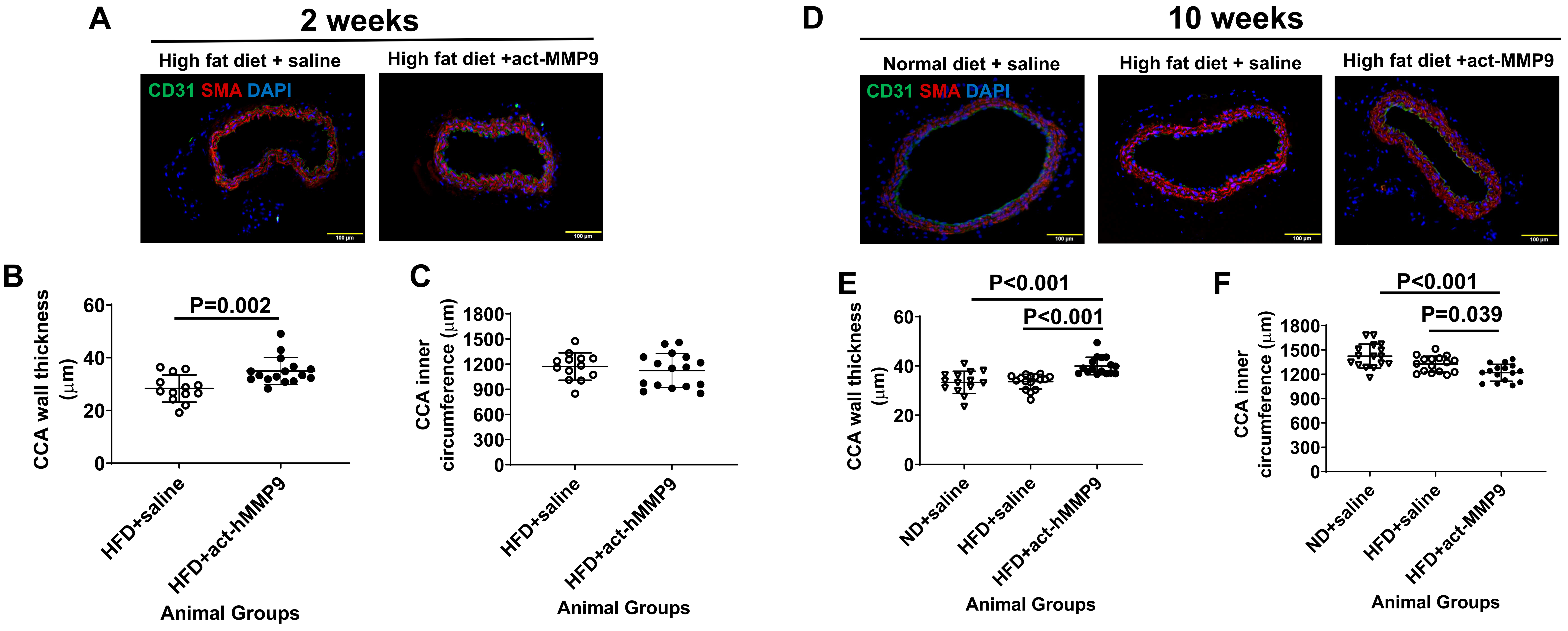

Supplemental Figure 2. **Wall thickness and inner circumference of common carotid arteries (CCAs) of KK mice.** **(A)** Fluorescence staining for CD31 and SMA expression to visualize morphology of CCAs in mice at 2 weeks after treatment. Quantification of wall thickness **(B)** and inner circumferences **(C)** of CCAs at 2 weeks after treatment. **(D)** Fluorescence staining for CD31 and SMA expression to visualize morphology of CCAs in mice at 10 weeks after treatment. Quantification of wall thickness **(E)** and inner circumferences **(F)** of CCAs in mice at 10 weeks after treatment. N = 13 or 16 CCAs from 7 - 8 animals for each data. Values are presented as means ± SD. Panels B and C: Independent T-Test; Panels E and F: One-way ANOVA.
