# Supplementary material for: Matrix metallopeptidase 9 contributes to the beginning of plaque and is a potential biomarker for the early identification of atherosclerosis in asymptomatic patients with diabetes
Source: Front Endocrinol (Lausanne). 2024 Apr 10;15:1369369. doi: 10.3389/fendo.2024.1369369 (PMC11039961; doi:10.3389/fendo.2024.1369369)
Supplement: Supplementary file 2 [file DataSheet_2.pdf]

Supplemental Table 1. qRT-PCR primers

Gene Name	Forward	Reverse	Amplication size (bp)
**GAPDH**	TCGACAGTCAGCCGCATCTTCTTT	ACCAAATCCGTTGACTCCGACCTT	94
**MCP-1**	CGCCTCCAGCATGAAAGTCT	AGGTGACTGGGGCATTGATT	110
**VCAM-1**	GGAGCTCTACTCATTCCCTAGA	CTAGGAACCTTGCAGCTTACA	93
**ICAM-1**	GTAGCAGCCGCAGTCATAAT	GGGCCTGTTGTAGTCTGTATTT	94

Supplemental Table 2. Antibodies used in Western Blot analysis

Primary antibody	Dilution	Category NO.	Company	2nd antibody	Dilution	Company
**Mouse anti-MCP-1**	1:400	sc-32771	Santa Cruz	Goat anti-mouse-IgG-HRP	1:1000	perkin Elmer
**Rabbit anti-VCAM1**	1:1000	13662S	Cell signaling	Goat anti-rabbit-IgG-HRP	1:4000	Cell signaling
**Rabbit anti-ICAM-1**	1:1000	4915S	Cell signaling	Goat anti-rabbit-IgG-HRP	1:4000	Cell signaling
**Rabbit anti-pERK1/2**	1:1000	4370S	Cell signaling	Goat anti-rabbit-IgG-HRP	1:4000	Cell signaling
**Rabbit anti-ERK1/2**	1:1000	9102S	Cell signaling	Goat anti-rabbit-IgG-HRP	1:4000	Cell signaling
**Rabbit anti-pP38MAPK**	1:1000	4511S	Cell signaling	Goat anti-rabbit-IgG-HRP	1:4000	Cell signaling
**Rabbit anti-P38MAPK**	1:1000	9212S	Cell signaling	Goat anti-rabbit-IgG-HRP	1:4000	Cell signaling
**Rabbit anti-pNFκB**	1:2000	MA5-15160	Thermo Fisher	Goat anti-rabbit-IgG-HRP	1:4000	Cell signaling
**Mouse anti-NFκB**	1:500	sc-8008	Santa Cruz	Goat anti-mouse-IgG-HRP	1:4000	Perkin Elmer
**Rabbit anti-GAPDH**	1:5000	2118S	Cell signaling	Goat anti-rabbit-IgG-HRP	1:5000	Cell signaling
